# Automated pipeline processing X-ray diffraction data from dynamic compression experiments on the Extreme Conditions Beamline of PETRA III

**DOI:** 10.1107/S1600576724004114

**Published:** 2024-07-04

**Authors:** Mikhail Karnevskiy, Konstantin Glazyrin, Yuelong Yu, Anshuman Mondal, Carmen Sanchez-Valle, Hauke Marquardt, Rachel J. Husband, Earl O’Bannon, Clemens Prescher, Anton Barty, Hanns-Peter Liermann

**Affiliations:** a Deutsche Elektronen-Synchrotron DESY Notkestraße 85 22607Hamburg Germany; b Institute of Mineralogy University of Münster Corrensstraße 24 48149Münster Germany; c Department of Earth Sciences University of Oxford OxfordOX1 3AN United Kingdom; d Lawrence Livermore National Laboratory 7000 East Avenue Livermore CA94550 USA; e Institute of Earth and Environmental Sciences University of Freiburg Hermann-Herder-Straße 5 79104Freiburg Germany; Australian Synchrotron, ANSTO, Australia

**Keywords:** high-performance computing, pipeline data processing, batch pipeline processing, high-pressure studies, diamond anvil cells, dynamic compression, X-ray diffraction, visualization, analysis

## Abstract

Presented and discussed here is the implementation of a software solution that provides prompt X-ray diffraction data analysis during fast dynamic compression experiments conducted within the dynamic diamond anvil cell technique. It includes efficient data collection, streaming of data and metadata to a high-performance cluster (HPC), fast azimuthal data integration on the cluster, and tools for controlling the data processing steps and visualizing the data using the *DIOPTAS* software package. This data processing pipeline is invaluable for a great number of studies. The potential of the pipeline is illustrated with two examples of data collected on ammonia–water mixtures and multiphase mineral assemblies under high pressure. The pipeline is designed to be generic in nature and could be readily adapted to provide rapid feedback for many other X-ray diffraction techniques, *e.g.* large-volume press studies, *in situ* stress/strain studies, phase transformation studies, chemical reactions studied with high-resolution diffraction *etc.*

## Abstract 2

Tools have been implemented for rapid X-ray diffraction data processing and analysis for dynamic compression experiments using a diamond anvil cell.

## 1. Introduction

Dynamic compression experiments employing diamond anvil cells (DACs) are a rapidly developing field exploring *e.g.* material properties and phase equilibria as a function of compression rate. DACs enable experiments over a wide range of pressures and temperatures, and provide access to small or medium strain rates inaccessible in shock/ramp compression studies. A piezoelectrically driven diamond anvil cell can pressurize matter at speeds reaching terapascals per second (Jenei *et al.*, 2019 ▸) along a controllable compression path. This maturing technique has demonstrated its significance across diverse scientific areas including materials and physical sciences and geoscience (Marquardt *et al.*, 2018 ▸; Husband *et al.*, 2021 ▸). The overall duration of a dynamic compression X-ray diffraction experiment can be from less than a second to many minutes for acquisition of image sequences. Correspondingly, the final number of signal-bearing 2D detector frames is typically large, ranging from several hundred to tens of thousands of images, collected, for example, along a tuneable compression ramp.

Dynamic compression experiments are often single shot and can yield highly variable almost non-deterministic results. Rapid data processing and analysis greatly benefit these experiments, providing prompt feedback to scientists. For short image sequences acquired over a few seconds, near-instant feedback on experimental success is highly desirable, enabling swift decisions on subsequent experimental runs. For longer-duration image sequences spanning many minutes, fast feedback on the experiment’s progress is equally crucial. The practice of allocating relatively short periods of beamtime to experiments at large-scale facilities (*e.g.* the European X-ray Free-Electron Laser facility in Germany, PETRA III at DESY in Germany, ESRF/EBS in France, APS in the USA, SPRING-8 in Japan *etc.*) additionally emphasizes the need for rapid data analysis and feedback to scientists to optimize the use of available beamtime.

Similar challenges are encountered by users of other experimental techniques, especially during *in situ* experiments where sample conditions are changing rapidly. The use of computation-intensive techniques, such as multiprobe tomography, ptychography and serial crystallography, is becoming increasingly prevalent at large-scale synchrotron facilities. These experiments use high-performance X-ray detectors that combine high frame rates and multi-megapixel images. The resulting data may saturate existing facility communication lines, often necessitating the sharing of powerful centralized computational resources instead of relying on the limited resources dedicated to each individual instrument. From the perspective of scientific computing, it is acknowledged that the use of data processing pipelines running on high-performance dynamically allocated computing (HPC) cluster nodes can significantly reduce the time required for analysis and increase the overall experimental throughput. A smartly designed pipeline can efficiently handle large amounts of data, extract valuable information and adapt easily to changing demands. Thus, automated pipelines offer numerous benefits, including quicker reduction of experimental data. They find applications in various fields such as tomography (Hintermüller *et al.*, 2010 ▸) and serial crystallography (Nakane *et al.*, 2016 ▸).

Here, we present a data processing pipeline that enables the rapid analysis of X-ray diffraction (XRD) data, enhancing feedback from dynamic compression experiments conducted on beamline P02.2 at PETRA III. The analysis pipeline is structured as a general experiment-agnostic data transport and analysis framework. We use it as a base upon which we implement a processing algorithm specific to P02.2. Certain features developed within the project are already accessible to the broader community working with XRD through the use of the *DIOPTAS* software (Prescher & Prakapenka, 2015 ▸) and its code extension. The pipeline itself transfers data from the instrument to the computing centre for processing and analysis as soon as they are collected. The corresponding processing results are returned from the computing centre to the beamline for visualization as soon as the calculations are complete. In this modular solution, while some aspects are specific to certain software environments of DESY, the critical components can be implemented with minimal effort at other large-scale facilities. Our computing platform is designed to enable an easy deployment and adaptation for use in other experiments at PETRA III or other synchrotron facilities.

## 2. Instrumentation on beamline P02.2

Dynamic DAC experiments conducted on P02.2 involve material compression along a predefined pressure–time (*P*–*t*) path. The sample material is loaded into a DAC and the sample chamber is pre-compressed between two diamond anvils, *e.g.* by means of screws in the DAC, to reach a starting pressure. Conventionally, the sample chamber is created by making a hole in a pre-indented steel or rhenium gasket, which is typically filled with a pressure-transmitting medium, the sample material and a pressure marker. For a more recent review of the DAC, its limits and applicability, we refer to the work of O’Bannon *et al.* (2018 ▸) and the references therein. For a more detailed description of dynamic compression experiments, we refer the reader to, for example, Jenei *et al.* (2019 ▸) and Méndez *et al.* (2020 ▸).

Dynamic compression in the DAC is driven by a piezoelectric actuator controlled by a voltage produced by a piezoelectric amplifier (1000 V, 7 A; Piezosystem Jena GmbH). The amplifier enhances the pre-programmed ramp voltage signal produced by a function generator (Agilent 33522B) and converts this signal into programmed extension and contraction of the piezoelectric actuator, thereby inducing a compression–decompression path or stress cycling. The duration of the ramps can be short, on the scale of a few microseconds, or long, spanning up to tens of minutes. The entire procedure of controllable compression along a ramp is synchronized with XRD detectors, such as the X-Spectrum Lambda 2M (Pennicard *et al.*, 2013 ▸, 2018 ▸). The Lambda 2M is a 2.3 megapixel GaAs hybrid pixel detector, with a pixel size of 55 × 55 µm, capable of running at 12 bits per pixel and 2 kHz without dead time due to readout. As introduced by Jenei *et al.* (2019 ▸), two or more Lambda 2M detectors can be triggered with a 250 µs delay with respect to each other, producing a synchronized 4 kHz data stream in 12-bit mode. During the process of writing this manuscript, the same detectors recently received new firmware, allowing data collection at a speed of 24 kHz with 1 bit per pixel. Individual data frames are decoded by the detector control computer (a PC) and compressed using the DEFLATE algorithm (Deutsch, 1996 ▸) before being transferred as a single or as a batch of NeXuS files (Könnecke *et al.*, 2015 ▸) via 10 GE (gigabit ethernet) links into the DESY centralized network-based storage system based on the General Parallel File System (GPFS) architecture developed by IBM (https://www.ibm.com/docs/en/STXKQY/gpfsclustersfaq.html). Files saved into the GPFS can be accessed either from the individual beamline PCs or from the computing nodes on the Maxwell HPC cluster at DESY (https://confluence.desy.de/display/MXW/Maxwell+Cluster).

## 3. Automatic pipeline for dynamic compression XRD experiments

The prompt processing of hundreds or thousands of images immediately after a single compression or decompression ramp experiment is crucial for the success of the experimental programme. As processing such a substantial amount of data manually in a reasonable time is impractical and sometimes impossible, we have developed an automatic pipeline to simplify the process of data analysis and decision making during the experiment.

The pipeline runs on central computing infrastructure located remotely from the instrument itself, specifically a dynamically allocated node of the Maxwell HPC cluster. The Maxwell cluster itself is available to all of DESY with over 50 000 cores of heterogeneous computing and multiple petabytes of high-performance data storage. A dedicated analysis node is automatically allocated upon the start of a beamtime session to ensure computing resource availability during an experiment. The typical configuration of the Maxwell nodes available for experiments is shown in Appendix *A*[App appa]. The pipeline reads the metadata stream created by the detector driver software, processes the individual files indicated by the metadata stream and stitches the individual modules of the Lambda 2M detector into a single 2D image. In our case, the original data from the Lambda 2M detector are saved into three separate data sets (file streams) corresponding to the number of Lambda 2M individual modules. This explains additional processing, namely the necessity of assembling signals from several detector modules into a full 2D picture for a single frame. The pipeline then applies a 2D mask to the full image and subsequently performs an azimuthal integration, converting the signal-bearing 2D X-ray diffraction frames into 1D diffraction profiles using pre-calibrated instrument-specific parameters. In the final processing steps, the pipeline stitches together all 1D diffraction profiles and saves them into a single output NeXuS file. If a raw 2D data set was split into several batches of NeXuS files, the pipeline takes this into account and the resulting output NeXuS file contains information about the entire data set featuring, at times, hundreds to tens of thousands of frames.

The pipeline and data processing capability are provided as a service for the PETRA III beamlines through a collaboration between the beamlines, the recently established scientific computing group (FS-SC), the experimental control group (FS-EC), the detector group (FS-DS) and the central DESY computing centre. These centrally coordinated groups contributed to the development of the data acquisition system, including the detector driver. They also participated in programming the data processing pipelines and software for post-processing, including visualization, with the intention of enabling redeployment and reuse at other instruments.

**Figure 1:**
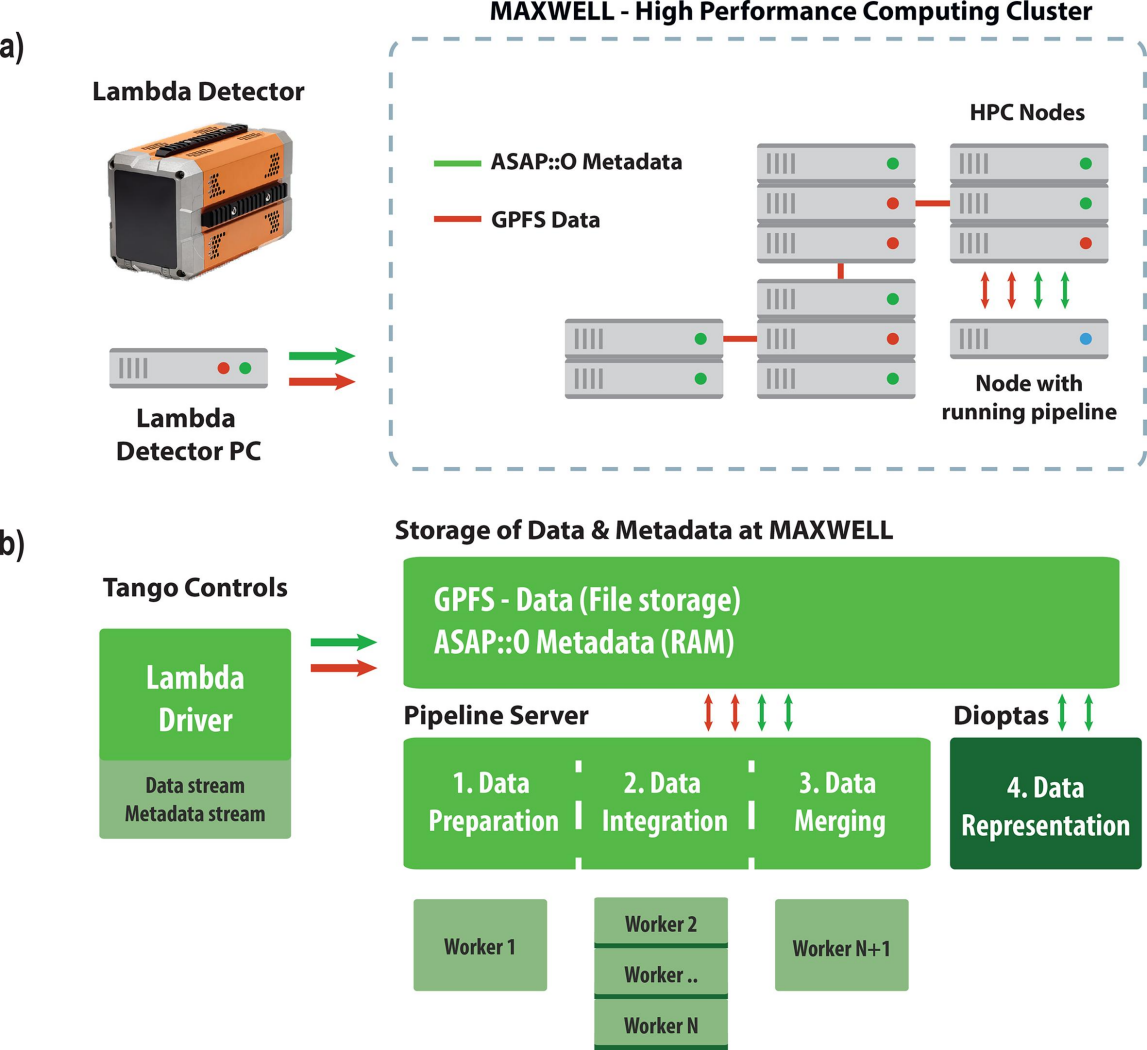
The operation of the data processing pipeline. (*a*) The initial step of data acquisition using the Lambda 2M detector, where a metadata stream is created and recorded within the distributed ASAP::O system. The data are then collected by the detector and stored in the form of NeXuS files, which are transferred to the GPFS storage within the HPC cluster. A separate processing node within the cluster is dynamically allocated for a specific beamtime session and hosts the pipeline process. (*b*) An illustration of how the driver of the Lambda detector creates a data stream in the form of NeXuS files and streams metadata containing their filenames via ASAP::O messages. This information can be accessed at different stages of the pipeline, numbered from one to three. The pipeline uses a configuration file and allocates several processes, defined as workers, utilizing multiple cores of the HPC node. Each worker performs a specific role, such as (1) data preparation, (2) integration using the *pyFAI* code (Ashiotis *et al.*, 2015 ▸) and (3) data merging. Coordination between the different workers is also done via ASAP::O metadata messages. The pipeline is designed to operate efficiently during data acquisition and saves time due to automatic data processing. The fourth and final step requires the use of the *DIOPTAS* software to view the processed data or initiate another round of reintegration at an HPC node when the allocated pipeline node becomes unavailable.

During the experiment, scientists can reprocess the data on an allocated node using a convenient web-based interface. After the end of an allocated beamtime session, scientists can analyse and reprocess their data using the *DIOPTAS* software package, either via a standalone private PC installation or via the installation at the Maxwell cluster nodes of DESY. The specific features of the pipeline and its operation are discussed in greater detail below. An overview of the data processing pipeline is presented in Fig. 1 ▸.

Several aspects of Fig. 1 ▸ are worth noting. The experimental control at PETRA III is based on the *Tango Controls* environment (https://www.tango-controls.org/), an open-source framework that is used at many large-scale facilities (*e.g.* DESY, ESRF/EBS, ALBA in Spain, SOLEIL in France, ELETTRA in Italy and FRM-II in Germany) as a supervisory control and data acquisition system. This environment plays a central role in driver development for detector control, in our case generating ASAP::O messages that provide information about the status of data acquisition.

Metadata streams containing information about the experiment and 2D detector frames are distributed using the ASAP::O high-performance decentralized streaming platform developed by the central IT department at DESY (https://asapo.pages.desy.de/asapo/; the code is available through https://gitlab.desy.de/asapo/asapo). This platform facilitates the streaming of raw data and metadata from various sources and utilizes the computational capabilities of its own cluster resources as its backbone. The ASAP::O system offers a C++ and Python application programming interface (API). Its architecture comprises servers responsible for communication between ‘producers’ and ‘consumers’, where the former generate both raw data and metadata and the latter store experimentally relevant information or participate in data processing. The system is highly customizable and can be adapted to the specific requirements of individual instruments. In our case, the detector driver plays the role of the ‘producer’ that sends a notification about the acquired data being saved. Individual elements of the pipeline-related workflow operate as both consumers and producers, as described below.

During dynamic compression experiments on the P02.2 beamline, the Lambda 2M drivers or *Tango* servers generate a stream of messages to the ASAP::O system when a data set or a portion of a data set is written to the data storage medium. Due to certain limitations concerning immediate write/read access of the NeXuS format based on HDF5 (Folk *et al.*, 2011 ▸; Könnecke *et al.*, 2015 ▸; https://www.hdfgroup.org/) and the fact that data sets may consist of several thousands of 2D data frames, it is necessary to divide the data sets into smaller chunks for effective data processing. After writing individual chunks of data to the GPFS, the detector driver sends the corresponding announcing messages.

The pipeline is initiated on an HPC node at the stage of detector driver initialization. A preconfigured setting file is applied that includes information about the allocated beamtime, service information on detectors used for data processing and configuration information for utilizing the available CPU cores in parallel operation. The pipeline code is stored centrally in a shared location within the GPFS storage and is executed with automatically configured access control list (ACL) rights. The pipeline is implemented in Python and utilizes Python virtual environments for operation on Maxwell nodes.

The application of the configuration file is only a first step for pipeline startup during a beamtime session. The next step involves two main processes: collection of a powder diffraction pattern of a calibrant, followed by calibration of the detector instrumental parameters (sample-to-detector distance and tilt). This calibration is then saved as a *pyFAI*-compatible PONI file. Additionally, a detector mask is created to identify any clusters of bad pixels on the detector, as well as areas with contaminated signals, such as diffuse scattering or diamond anvil Bragg diffraction signal. Since multiple detectors may be in use, this information must be collected for all detectors. During an experiment, the calibration and mask can be changed, and the pipeline has a user-friendly interface to update these parameters. To facilitate this and other tasks, such as pipeline troubleshooting, the pipeline also includes a web service based on Flask (https://flask.palletsprojects.com) using React (https://reactjs.org/) for interaction with the beamline operator. This web service allows for fast and convenient reconfiguration of the *pyFAI*-based integration parameters to be applied by the processing backend. Additional information about the web service functionality and code dependencies can be found in Appendix *B*[App appb]. After the configuration, the pipeline will use specially spawned processes defined as ‘workers’ for the various tasks, denoted by numbers 1–3 in Fig. 1 ▸.

During operation, the pipeline continuously retrieves data acquisition events from the ASAP::O metadata storage. Data from a single or multiple detectors and even from individual detector modules can be processed at the same time, taking advantage of multicore processing. Different detectors may have their own specific properties. For the Lambda 2M detectors the information is split into three files, each corresponding to an individual module. If the data (full acquisition or a chunk of an incomplete acquisition) are readable as tested by the process of Worker 1, the latter stitches the individual modules into a single 2D frame and sends a message or stream of ASAP::O messages to inform the workers of the subsequent Step 2 that new data are available and ready for further processing. These messages contain information on a single frame or a small batch of frames to enable parallel processing on an image-based level. Other detectors used at large-scale facilities may have data storage structures of variable complexity, and thus the pipeline’s modular approach allows tailoring and adjustment for individual detectors and individual beamline requirements.

In Step 2, the data undergo azimuthal integration using the prepared instrumental model and the pixel mask. This process can be efficiently parallelized across several worker processes, as indicated in Fig. 1 ▸. Upon completion of these tasks, the workers of Step 2 inform the workers of Step 3 where the 1D profile information is consolidated into a single NeXuS file, corresponding to a specific acquisition from a specific detector, as determined by the ASAP::O message content.

If any adjustments of integration parameters are necessary, the beamline operator can change the pixel mask and re-integrate the data. This can be done during the beamtime session by submitting a *Slurm* job (Yoo *et al.*, 2003 ▸) at the pipeline node via the web interface, which will run on the allocated compute node and will not interfere with ongoing data analysis. In this scenario, the previous result of integration will not be overwritten, but the output filename will be adjusted and made unique to reflect the new data processing.

In Step 3, the corresponding worker will complete the automatic pipeline-driven data processing. At this point we obtain a single file or a batch of NeXuS files with 1D diffraction data sets written as a function of frame number. Typically, the data are written as a function of 2θ°, but the pipeline web interface allows processing using the *Q*-range option. It is desirable that the data are inspected by the experimenters as quickly as possible at the end of the data acquisition to identify any possible issues, for example requiring changes in detector masks *etc.*, so that these can be corrected and fed back into the operating pipeline. Our development includes a viewer of results to facilitate monitoring of the data processing for this purpose.

**Figure 2:**
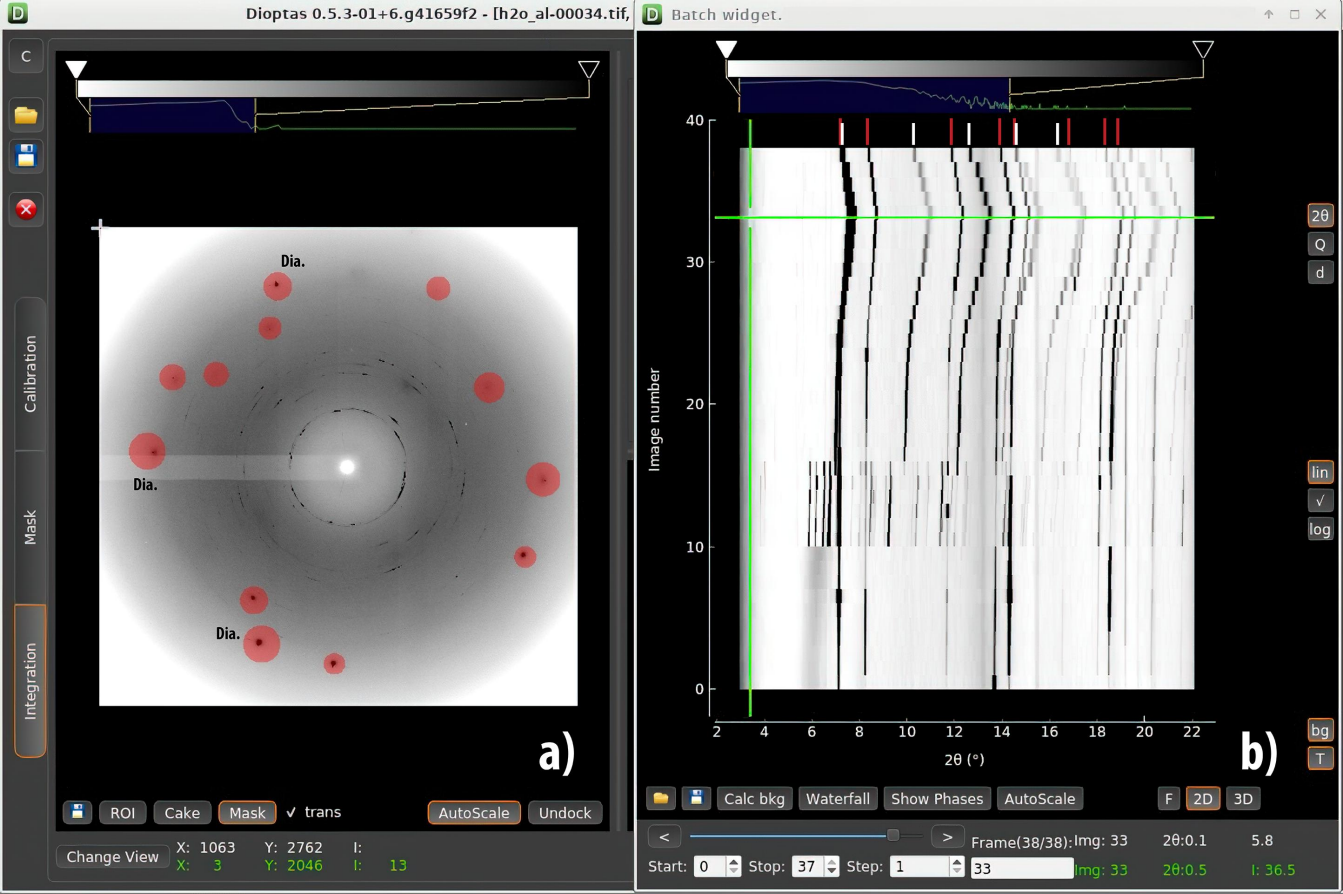
(*a*) A *DIOPTAS* window screenshot with (*b*) an overlay of the *Batch View* widget. The interface of the widget will be very familiar to the high-pressure community using *DIOPTAS*. The widget functionality supports various features, including integration of a data set, calculation and subtraction of the background for 1D patterns, visualization of different phases in the form of vertical lines for the purpose of phase identification, and image export for publication purposes. The 2D diffraction pattern in panel (*b*) shows water and aluminium powder loaded into a DAC with an aperture of approximately 64° (Boehler–Almax diamonds). The wavelength of the incident radiation was 0.2900 Å. The red spots indicate the Bragg reflections of the diamond anvils. The heatmap plot in panel (*b*) is generated by the code and illustrates the full data set, collected using a PerkinElmer XRD 1621, of a slow dynamic compression experiment conducted using a membrane DAC. There is a clear appearance of new lines upon compression, indicating the formation of ice VI (visible for a 2θ range of 6–7.5° and frame numbers 10–16) which transforms later to ice VII. The highest pressure indicated by the aluminium powder was 20.2 (2) GPa (Holian, 1986 ▸). The horizontal green line indicates the frame with the highest pressure. Vertical ticks above the heatmap indicate signal positions for the ice VII (red) and aluminium (white) phase reflections, respectively. Additional information about the *DIOPTAS* software and the new code’s capabilities can be found in Appendix *C*[App appc]. The screenshot corresponds to the early development version of *DIOPTAS*, Version 0.5.3.

The resulting data are displayed by a separate viewer so that scientists can draw conclusions regarding the efficiency and success of individual dynamic runs (Step 4 of Fig. 1 ▸). To aid in data visualization, new code (the *Batch View* widget) was added to *DIOPTAS* (available starting from the development branch of Version 0.5.3) that enables rapid data examination. In Fig. 2 ▸, we show a screenshot from the *DIOPTAS* main window and the *Batch View* widget which is frequently utilized by groups employing either slow, quasi-static or fast dynamic compression. Supplementary to the data from the fast Lambda 2M detectors shown in the next section, this example demonstrates processing of PerkinElmer XRD 1621 detector data. The implementation of new functionality within *DIOPTAS* enables batch 1D integration on both standalone PCs and cluster nodes for a wide range of detector image formats, which can be accessed using the *fabio* Python package (Knudsen *et al.*, 2013 ▸).

## 4. Application

Dynamic compression experiments are widely applied in various materials systems and contribute to multiple scientific disciplines, such as applied and functional materials, or those relevant to geo- and planetary sciences. Here we present examples of the application of our pipeline to dynamic compression studies that investigate the stability of planetary ices, with the example of ammonia–water mixtures, and stress and strain distributions of polymineralic samples during cycling loading.

### 4.1. Dynamic compression of ammonia–water mixtures

Planetary ices (H_2_, He, H_2_O, NH_3_, CH_4_ and mixtures thereof) are not only typical examples of low-*Z* molecular compounds but also important building blocks of planetary bodies, from comets to the interiors of icy moons and large solar (Uranus and Neptune) and extra-solar (mini-Neptune exoplanets) icy bodies (Journaux *et al.*, 2020 ▸; Fortney & Nettelmann, 2010 ▸; Borucki *et al.*, 2010 ▸, 2013 ▸; Rivera *et al.*, 2005 ▸; Batalha *et al.*, 2013 ▸). The high-pressure–temperature behaviour of planetary ices is thus critical to understanding the internal dynamics of water-rich planetary bodies. Dynamically driven compression experiments in diamond anvil cells are opening new avenues to explore these low-*Z* and often highly reactive compounds, as the rapid compression of the samples minimizes the risk of reactions with the gasket material and/or the diffusion of the sample into the diamond anvil, particularly at high temperatures (McMahon *et al.*, 2012 ▸). The approach has been validated in compressional studies of ammonia dihydrate (ADH, 1:2 NH_3_–H_2_O) (Mondal *et al.*, 2023 ▸), which is one of the three stable ammonia hydrates formed in the NH_3_–H_2_O system (Fortes & Choukroun, 2010 ▸). Here, we present an experimental study of the stability and compressibility of ammonia hemihydrate (AHH, 2:1 NH_3_–H_2_O), an ammonia-rich hydrate (Wilson *et al.*, 2012 ▸), and its mixture with H_2_O ice VII. This assembly may result from the decomposition of the H_2_O-rich ammonia hydrates ADH or ammonia monohydrate (1:1 NH_3_–H_2_O) (Wilson *et al.*, 2012 ▸, 2015 ▸).

We have employed a dynamic DAC (dDAC) equipped with 0.2 mm diameter diamond culets to compress the system continuously up to a pressure of 71 GPa at room temperature. The starting material for the experiments was an NH_3_–H_2_O solution containing 25 wt% NH_3_. In this study we used gold as the pressure marker. During the compression, XRD images were collected using a monochromatic synchrotron X-ray beam of 25.59 keV (wavelength = 0.4845 Å) with a focal spot size of 3 × 8 µm^2^ FWHM on the Extreme Conditions Beamline P0.2.2 at PETRA III (Liermann *et al.*, 2015 ▸). Two GaAs Lambda (2 × 2.3 megapixel) detectors (Pennicard *et al.*, 2013 ▸, 2018 ▸) were used for fast time-resolved X-ray data collection. A total of 800 diffraction patterns were collected during the compression experiment with a typical acquisition time of 1 s per pattern. Diffraction images were integrated using the data pipeline described above to obtain 1D diffraction patterns as a function of 2θ and then summarized as a heatmap image using the *DIOPTAS* software.

**Figure 3:**
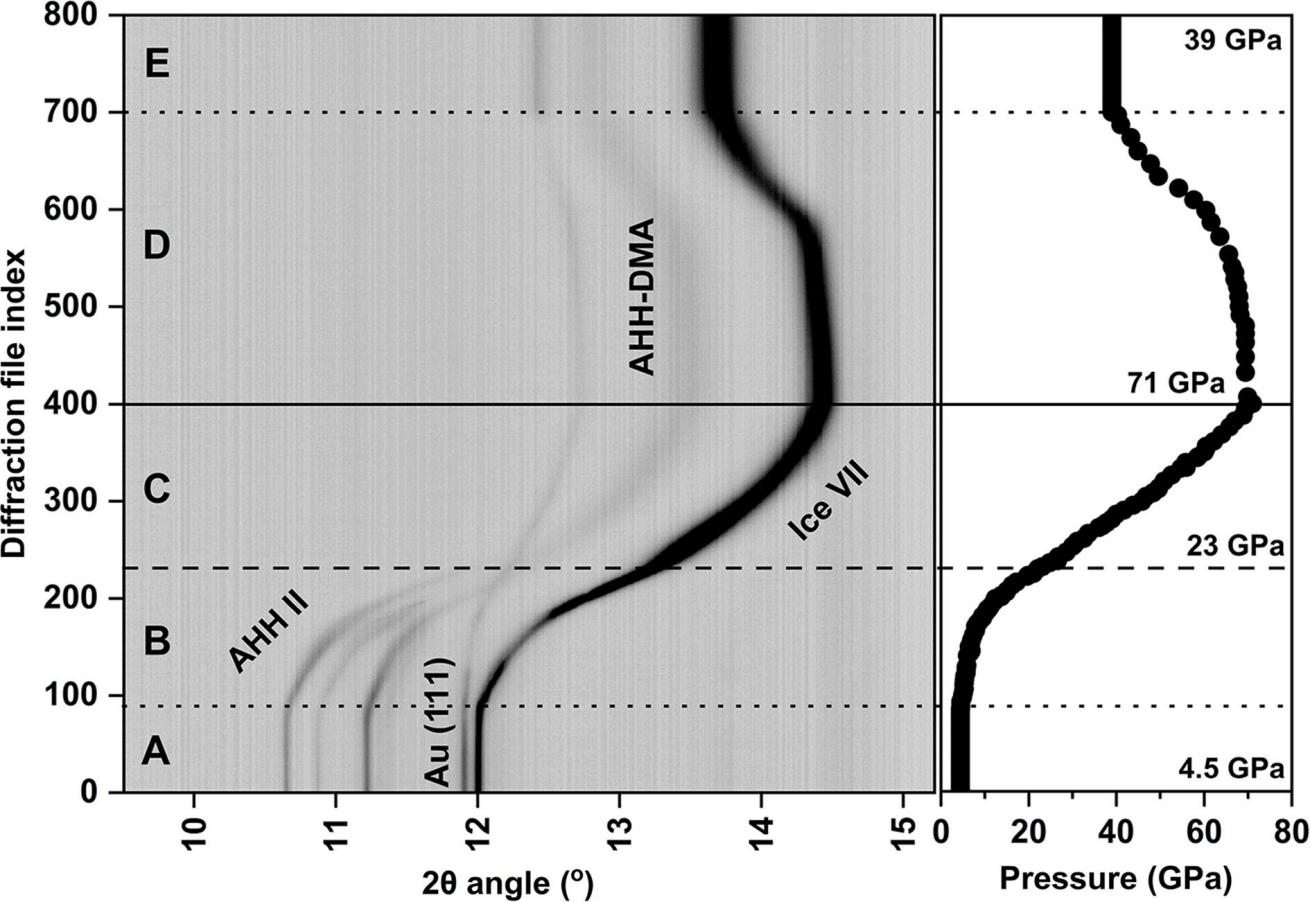
A heatmap combining 800 integrated patterns, showing the evolution of the compression–decompression ramp run. The panel shown on the right illustrates the pressure evolution. Using the letters A to E, we indicate different important steps of the compression ramp. Horizontal lines of various styles indicate selected pressure points and can be used as guides for the eye. From bottom to top, they highlight the points of 4.5 GPa (image 90), 23 GPa (image 233), 71 GPa (image 400) and 39 GPa (image 700). Steps A and E correspond to the starting and final steps of compression, respectively (the voltage applied to the piezoelectric drive is equal to zero). At the beginning of step B, the force applied by the piezoelectric drive becomes sufficient to change the pressure within the sample chamber. The transition from AHH II to AHH-DMA is observed at around 23 GPa. Within the region B–C the pressure rises to the limit determined by the maximum voltage of the voltage amplifier connected to the piezoelectric drive. Within region D, the voltage applied to the piezoelectric drive decreases, leading to decompression within the sample chamber. The pressure is calculated from the 111 reflection of Au, which is employed as a pressure marker. Error bars of pressure determination should be considered to be equal to the symbol size or smaller.

All diffraction images collected during one single compression–decompression run are illustrated in Fig. 3 ▸. Our analysis shows that the 25 wt% NH_3_–H_2_O solution fractionally crystallizes into a mixture of AHH (phase II) and ice VII after a pre-compression to 4.5 GPa. Three characteristic reflections arising from the AHH II monoclinic crystal structure, *i.e.*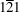, 102 and 023, can be observed at the beginning of the compression ramp at 2θ angles of 10.65, 10.87 and 11.22°, respectively. Upon compression, the three reflections from AHH II merge into a single broad reflection at pressures above ∼23 GPa, with the latter attributed to AHH disordered molecular alloy (AHH-DMA; body-centred cubic structure) as proposed by Ma *et al.* (2012 ▸). No further changes in the crystal structure were observed up to the highest pressure of 71 GPa. This and similar experiments emphasize the importance of rapid data analysis shortly after the compression run as it is essential for evaluating the success of the experiment. The results of our work will be published in greater detail elsewhere.

### 4.2. Dynamic compression oscillation of multiphase mineral assembly

Seismic data provide the most direct information about the inaccessible deep interior of Earth and dynamic processes inside our planet. In order to interpret this information accurately, we require knowledge of the elastic properties of Earth’s constituent minerals, as well as an in-depth understanding of their elastic and plastic interactions under oscillating strain, *e.g.* as induced by propagating seismic waves. Time-resolved XRD experiments using the dDAC allow us to measure and characterize elastic properties of minerals, providing a promising way of constraining elasto-plastic interactions.

We see more and more exciting applications of time-resolved experiments using the dDAC with an oscillating load around a mean pressure with continuous measurement of XRD. The realization of the potential of the method was demonstrated for (Mg,Fe)O at 300 K (Marquardt *et al.*, 2018 ▸) and more recently tested at high temperatures (Trautner *et al.*, 2023 ▸). Stress cycling experiments and time-resolved continuous compression experiments are very rapid ways of measuring compressibility (Trautner *et al.*, 2023 ▸; Wang *et al.*, 2023 ▸). They are also ideally suited for compression studies at high temperatures (Trautner *et al.*, 2023 ▸) where experiments become unstable with time in the resistively heated dDAC. The approach promises a continuous mapping of the elastic bulk modulus in pressure–temperature–composition space, but requires the analysis of a large amount of collected XRD data.

Pressure oscillation experiments, in principle, also enable the measurement of distributions of stress and strain throughout polymineralic samples under cyclic loading. Such measurements tackle one of the major limitations in our current understanding of the seismic properties of mantle rocks, which relies on employing averaging schemes (Thomsen, 1972 ▸) that may lead to intrinsic uncertainties of up to several percent. Considering various applications, pressure oscillation experiments might also enable measurements of the frequency dependencies of the compression behaviour which might occur via dissipative processes. For example, seismic attenuation can occur when processes take place on the same timescale (or frequency) as the pressure (stress) variations induced by a passing seismic wave (frequencies of about 0.001–100 Hz). In single-phase materials, this includes the possible movement of twin walls in tetragonal CaSiO_3_ or a possible effect of low-spin/high-spin clustering in (Mg,Fe)O (Carpenter & Zhang, 2011 ▸) under conditions of planetary interiors. In two-phase regions, this includes element diffusion processes (Li & Weidner, 2008 ▸; Li, 2010 ▸; Perrillat *et al.*, 2016 ▸). Pressure oscillation experiments have the potential to reveal dissipation frequencies and to characterize the distribution of stress and strain during cyclic loading. The capability of running oscillating load experiments in the dDAC over a wide range of frequency, strain and temperature conditions, coupled with the time-resolved monitoring of the sample, will enable exploration of various natural processes in great detail.

**Figure 4:**
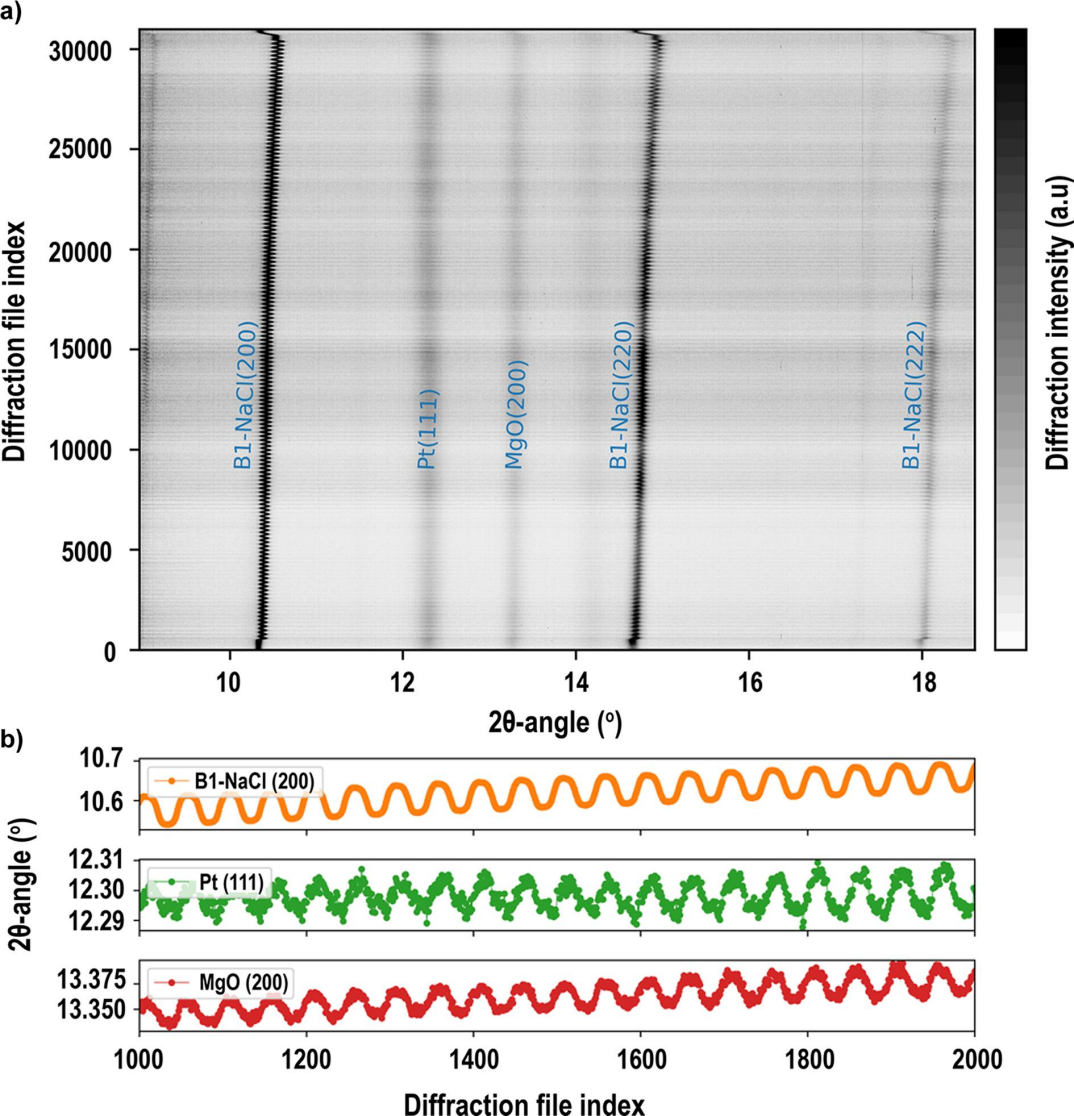
Data representation based on 31 000 diffraction images, showing the evolution of sample compression including NaCl, Pt and MgO. (*a*) A heatmap representing the data with indication of the individual diffraction lines. The pressure at the start of oscillations in this experiment was around 5 GPa, with pressure cycling amplitudes estimated as 0.5–1 GPa. The pressure oscillations are most clearly observed in the NaCl diffraction lines. A slight increase in pressure, on top of the pressure oscillations, is observed throughout the experiment, and this should be attributed to a deformation of the DAC gasket material yielding to the conditions of cycling stress. (*b*) A magnification of selected 2θ ranges to illustrate the cycling of reflection positions between images 1000–2000. (Image provided by Biao Wang, University of Oxford.)

We tested the capabilities of using the dDAC to characterize the response of polymineralic samples to cyclic pressure oscillations and to probe possible time dependencies. In the experiments we loaded powder mixtures of MgO and NaCl along with platinum into a symmetric DAC equipped with 0.3 mm culet diamonds. The DAC was coupled to a piezoelectric actuator as implemented on the Extreme Conditions Beamline P02.2 at DESY (Jenei *et al.*, 2019 ▸). We used the dDAC setup to pre-compress the samples and apply cyclic loads with different frequencies and amplitudes of the oscillations covering typical seismic frequencies. XRD images were collected using two GaAs Lambda 2M detectors. Our results are to be published separately, but in Fig. 4 ▸ we show a typical heatmap plot constructed from 31 000 diffraction images. Here, the sample was compressed to about 5 GPa and subjected to sinusoidal pressure cycles at a frequency of 1 Hz and oscillation amplitudes approximating to ±0.5–1.0 GPa. Images were collected continuously with a single image col­lec­tion time of 50 ms and 2000 frames per data acquisition file.

## 5. Conclusions

As a response to the challenges of fast data analysis of dynamic compression experiments, we have developed a software solution that improves the throughput and success rate of demanding high-pressure experiments, *e.g.* involving dynamic compression ramps or oscillation ramps, with application to fundamental and planetary sciences. It extends the capability of the Extreme Conditions Beamline P02.2 at PETRA III and simplifies the steps of data preparation, data conversion, data integration and visualization of experimental results during or shortly after data acquisition. The software performs calculations either remotely, using DESY HPC resources with robust feedback, or on a private PC as an extension of the *DIOPTAS* software suite for data visualization or post-processing.

Using the examples of ammonia mixtures and mineral assembly compression we have illustrated the importance of modern scientific computing for state-of-the-art processes of experimental evaluation.

The example code for the P02.2 pipeline is available at https://gitlab.desy.de/fs-sc/integrationworker.

Our processing platform is available to general users of the beamline for both on-site and off-site execution. We intend the software to be modular and anticipate that our open-source solution will be extended or applied on other beamlines at PETRA III, as well as at other large-scale facilities, in order to optimize the use of allocated beamtime.

## Figures and Tables

**Figure 5:**
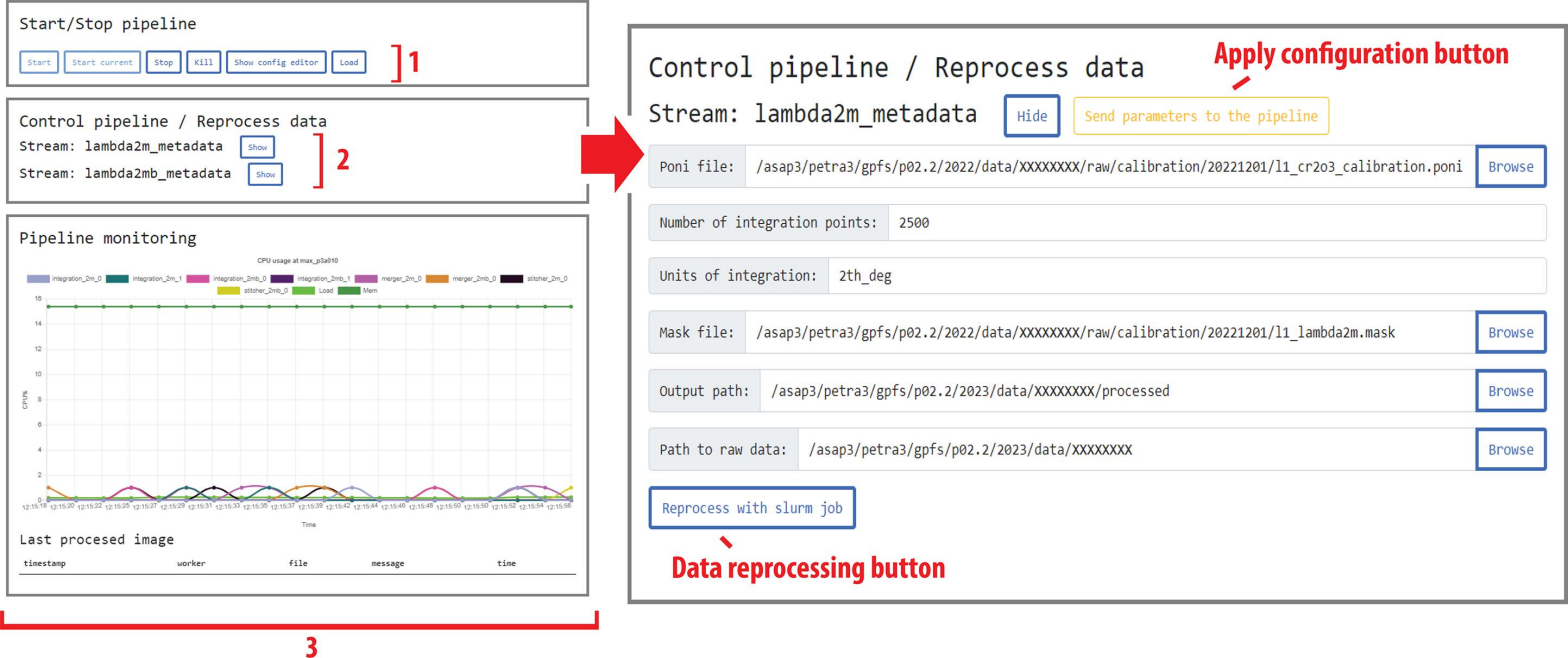
Screenshots illustrating the web interface used for control of integration parameters. The roles of the indicated fields are discussed in the text.

**Figure 6:**
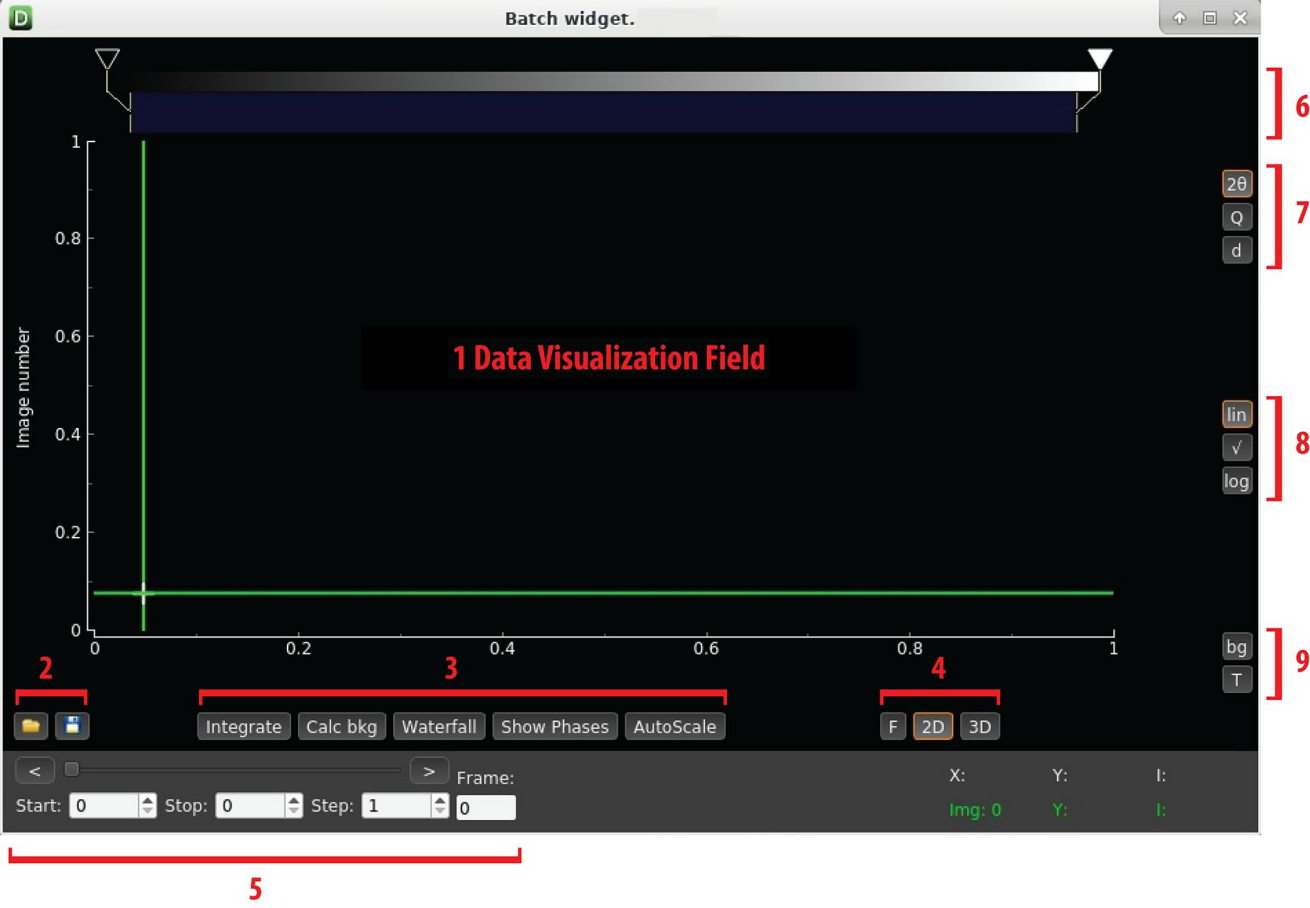
A screenshot illustrating the interface of the *Batch View* widget. The roles of the fields indicated from 1 to 9 are discussed in the text. The picture corresponds to *DIOPTAS* Version 0.5.3 as installed at the MAXWELL cluster under Linux. With versions 0.5.8 and 0.5.9, *DIOPTAS* has undergone a style change, but the layout remains similar and recognizable.

## Appendix A Supplementary information about MAXWELL nodes and ASAP::O

The typical configuration of MAXWELL nodes available for experiments at PETRA III and used during experiments on P02.2 has 40 CPU cores and 500 GB RAM using a *CentOS* Linux distribution (Version 7 at the time of writing). All nodes have access to the GPFS (https://www.ibm.com/docs/en/STXKQY/gpfsclustersfaq.html) and special ACLs are allocated for a specific beamtime session for safety reasons.

The transfer of data between the detectors and various components of the data processing pipeline can be facilitated by a streaming platform called ASAP::O. Developed at DESY, the platform is supported as a service by the central IT department. ASAP::O comprises three main components: a central service that provides the core functionality, and producer and consumer APIs for ingesting and retrieving data from the system, respectively.

Data organization within ASAP::O follows a hierarchical structure. It begins with the beamline identifier followed by beamtime identifying numbers (IDs). Each detector corresponds to its own ASAP::O data source, with modular detectors potentially utilizing a sub-source concept within a parent data source. For example, three modules of a Lambda detector may send data to a single data source with three sub-sources. Each data source can emit multiple data streams, each identified by a unique name within that specific data source. These streams consist of smaller entities called messages, which are flexible in content to accommodate a wide range of use cases. Data are retrieved from ASAP::O on request from a client or ‘consumer’.

ASAP::O is able to stream not only file names but also image data. In the future, one approach for sending image data will be to use compression, allowing for a writing speed of 200 to 600 images per second. Another approach is to send uncompressed data, which can reach a sustained rate of 500 images per second using the current setup (Lambda 2M), though this is limited by the 10 GE network between the server PCs and the Maxwell cluster where ASAP::O is running.

## Appendix B The pipeline and its control web interface

The code dependencies for the pipeline include *DIOPTAS* and its relevant dependencies (Prescher & Prakapenka, 2015 ▸). The web interface also utilizes Flask (https://flask.palletsprojects.com) and Bootstrap (https://getbootstrap.com/) for the back end and React (https://reactjs.org/) for the front end. The interface allows the beamline operator easily to start and stop automatic pipeline operation, to provide and change instrumental models, and to apply pixel masks for the integration of 2D diffraction patterns into 1D profiles. The front end allows for easy file selection through web-based navigation. It also provides control for the number of bins used in azimuthal integration, the ability to start *Slurm* (Yoo *et al.*, 2003 ▸) jobs for data reintegration, and other functionalities. The web interface of the pipeline can be opened from various PCs, which also helps with technical support and troubleshooting. Below we show the most important elements of the pipeline inteface.

Fig. 5 ▸ presents screenshots of the web interface used for controlling the pipeline and its processes. The interface is designed for ease of use and most of its features are self-explanatory. For example, field 1 is responsible for loading experiment-specific configurations, including information on starting processes for data preparation, integration and output, as well as interprocess communication (via ASAP::O messages).

Crucially, even if the processes are started, data processing cannot occur without initialization of a calibration file, pixel mask and other supplementary information. For the sake of clarity, some additional fields that allow for fine-tuning the integration have been omitted in the representation. Field 2, as demonstrated in the right-hand panel of Fig. 5 ▸, is utilized to apply this necessary information.

Field 3, also shown in Fig. 5 ▸, provides real-time updates on the status of pipeline processes and serves multiple purposes. It displays the current CPU load of specific processes, providing insight into the state of data set processing. Additionally, it serves as a clear indication of process crashes, which is essential during development and troubleshooting phases. Additional information in the case of problems can be found in the pipeline logs, which are easily accessible from the interface, but these details are omitted here for brevity.

## Appendix C *DIOPTAS* and the *Batch View* widget for data visualization

*DIOPTAS* is developed under the open-source initiative (Prescher & Prakapenka, 2015 ▸) and it has acquired a large scientific user base, including those working at high pressure. We extended the code of *DIOPTAS* to be able to visualize data prepared by the pipeline and to reprocess data on a standalone computer, and this extension is dubbed the *Batch View* widget.

The controls of the *Batch View* widget shown in Fig. 6 ▸ resemble those of *DIOPTAS*. The numbers 1 to 9 indicate the typical fields used by an operator. Field 1 corresponds to the data visualization field. Field 2 contains buttons either to open data or to export to file for publication purposes (graphic image file format) or for further processing (NeXus file format). Considering data opening, one can open either a data set prepared by the pipeline or a sequence of 2D frames saved in a format compatible with the *DIOPTAS* main code, relying on the *fabio* Python library (Knudsen *et al.*, 2013 ▸).

In field 3, the data can be integrated or processed further, *e.g.* for a background correction step. For example, the ‘Waterfall’ button initiates a waterfall-like plot of 1D data patterns within the main window of *DIOPTAS* and the ‘Show Phases’ button overlays the synthetic signal from various structures or phases loaded by the main *DIOPTAS* window for the purpose of signal identification.

The buttons located in field 4 are used for switching between different views: the ‘F’ button shows a list of loaded frames or data, the ‘2D’ button switches on the 2D heatmap view of processed data and the ‘3D’ button shows the same data in the form of a 3D mesh.

The controls in field 5 can be used to navigate through images. If possible, the *Batch View* widget can identify correlations between data shown in the data visualization field and the raw 2D images, allowing for selection of a 1D profile of interest within *Batch View* and inspection of the corresponding 2D pattern in the main window of *DIOPTAS*.

Data visualization is also dependent on the state of field 6, which controls the colour representation and contrast of 2D heatmaps. Field 7 allows for switching between different data representations, including 2θ, *d* spacing and *Q*-range options. Field 8 controls data scaling for 2D or 3D representations, with options for linear, square root and logarithmic scales. Finally, the buttons in field 9 can optimize data visualization, such as the ‘bg’ button, which switches on/off between raw data and background-subtracted views, and the ‘T’ button, which synchronizes *Batch View* with the lower and higher *x*-axis bounds of the main *DIOPTAS* window 1D pattern view.
